# Parents’ experiences of the financial and employment impacts of their child receiving end-of-life care: a national qualitative study

**DOI:** 10.1186/s12904-025-01796-1

**Published:** 2025-06-04

**Authors:** Laura Barrett, George Peat, Emma Victoria McLorie, Helen Weatherly, Sebastian Hinde, Gabriella Lake Walker, Jane Noyes, Sam Oddie, Chakrapani Vasudevan, Richard G. Feltbower, Bob Phillips, Catherine Hewitt, Richard Hain, Gayathri Subramanian, Andrew Haynes, Fliss E. M. Murtagh, Julia Hackett, Lorna Katharine Fraser

**Affiliations:** 1 The Paediatric Palliative Care Research Group, Department of Health Sciences, Faculty of Science, University of York, Rowntree Building Heslington, York, YO10 5DD UK; 2 Department of Social Work, Education and Community Wellbeing, Northumbria University, Newcastle upon Tyne, UK; 3 School of Healthcare, Faculty of Medicine and Health, University of Leeds, Leeds, UK; 4 Centre for Health Economics, University of York, York, UK; 5 Parent Advisory Panel Member, York, UK; 6 School of Medical and Health Sciences, Bangor University, Bangor, UK; 7 Bradford Hospitals National Health Service Trust, Bradford, UK; 8 Leeds Institute for Data Analytics, School of Medicine, University of Leeds, Leeds, UK; 9 Child Health Outcomes Research at Leeds (CHORAL), University of Leeds, Leeds, UK; 10 Centre for Reviews and Dissemination, University of York and Hull-York Medical School, York, UK; 11 Consultant in Paediatric and TYA Oncology, Leeds Children’s Hospital, Leeds, UK; 12 York Trials Unit, Department of Health Sciences, University of York, York, UK; 13 Noah’s Ark Children’s Hospital, Heath Park, Cardiff, UK; 14 Royal Manchester Children’s Hospital, Manchester Foundation Trust, Manchester, UK; 15 Wolfson Palliative Care Research Centre, Hull York Medical School, University of Hull, Hull, UK; 16 Cicely Saunders Institute, Department of Women’s and Children’s Health, King’s College London, London, UK

**Keywords:** Paediatric end-of-life care, Paediatric care services, Palliative care, Qualitative research, Guidelines, Financial support, Employment

## Abstract

**Background:**

Bereaved parents are at higher risk of poor mental and physical health outcomes than people bereaved under other circumstances. These challenges are exacerbated by the continued effects on parents’ working lives and the financial strain of a child receiving end-of-life care. There has been very little recent research of parents’ experiences of these impacts. Analysis of data from the second workstream of a national research programme on end-of-life care for infants, children and young people (ENHANCE) aimed to understand parents’ experiences of the impact on their finances and working lives while their child received end-of-life care.

**Methods:**

A multi-site qualitative study using in-depth interviews with bereaved parents, analysed using thematic analysis. Recruited through NHS sites, children’s hospices and via the social media of third sector organisations.

**Results:**

Forty-two interviews with 55 parents were conducted (Fathers = 16, Mothers = 39), representing 44 children. Four themes were developed: (1) The added cost burden; (2) Pressures of juggling work; (3) Accessing support; and (4) Financial impacts continue after a child dies.

**Conclusions:**

Financial hardship is a known consequence of having a child with a life-limiting condition, especially at the end of life, and adds considerable stress to an already painful situation, with the aftermaths continuing into bereavement. The impact is exacerbated by parents’ need to reduce work so they can spend time caring for their dying child, leaving families in a financially and emotionally vulnerable position. There needs to be a consistent approach to immediate practical support from healthcare providers; a review of benefit system delays and the abrupt stopping of Disability Living Allowance; and the development of tailored employment support for parents to remain in or rejoin the workforce.

**Supplementary Information:**

The online version contains supplementary material available at 10.1186/s12904-025-01796-1.

## Key messages

*What is already known on this topic*

*What this study adds*

*How this study might affect research*,* practice or policy*

• Bereaved parents are at higher risk of poor mental and physical health outcomes than people bereaved under other circumstances.

• There has been little recent research on the impacts on a family’s employment or the financial costs of caring for a child at the end of life.

• The direct financial costs to a family of a child receiving end-of-life care are significant and add considerable stress to an already painful situation.

• The impacts of additional costs are compounded by parents needing to balance time working with caring for their dying child, and this leaves families in a financially and emotionally vulnerable position.

• Parents lack guidance and support to navigate government financial support and charity funded support for which they may eligible.

• A proactive policy that is consistently applied is required from healthcare providers, to ensure all families with a child receiving end-of-life care are immediately supported practically and financially.

• The Government should review delays in processing of benefit applications, the immediate stopping of Disability Living Allowance when a child dies, and consider a transition to bereavement support payments.

• There is a need for improved guidance for employers to provide support which is tailored to the specific needs of those parents caring for a dying child and bereaved parents.

**Supplementary Information:**

The online version contains supplementary material available at 10.1186/s12904-025-01796-1.

## Background

Every year in the United Kingdom (UK), the parents of over 4,000 infants and children face the devastating reality of their child dying [1–5]. These bereaved parents are at higher risk of poor mental and physical health outcomes than people bereaved under other circumstances [6, 7]. These challenges are exacerbated by the impacts on the family’s working lives and financial costs of caring for a child at the end of life [8]. 

Before their child dies, families may already be in a financially precarious situation having faced the additional costs of caring for a seriously ill child [9, 10]. Children with complex and life-limiting conditions are living longer with more uncertain prognoses [11], and are often reliant on medical technologies at home for many years which has important social and economic consequences [12, 13]. A recent survey (2024) estimating the costs of having a disabled child, found that in most households caring responsibilities meant one parent had to give up a job or reduce their hours, resulting in an average reduction in annual household income of £21,174 [14]. 

The direct financial impacts of a child dying was first reported in the UK literature more than 20 years ago; [9, 15] however there is no recent evidence examining the costs, employment implications and access to financial support currently experienced by parents of a child at the end of life [8]. While there have been several changes to how the government supports families with a terminally ill child, it is still the case that there is an immediate loss of some benefits upon the child’s death. One area where there has been some progress in recent years, is the Children’s Funeral Fund For England, established in 2019 covering basic burial or cremation fees, similar schemes exist in the other nations of the UK [16, 17] but they do not cover all aspects of funeral costs for families.

This paper reports an analysis of data from the second workstream of a national research programme on end-of-life care for infants, children and young people (ENHANCE) [1]. The results of other analyses from this workstream have been published elsewhere [18, 19]. End-of-life care refers to care and support for children and their families in the last days, weeks, months or year of life [20, 21]. This analysis aimed to understand parents’ experiences of the impact on their finances and working lives while their child is receiving end-of-life care.

## Methods

These data were collected during a multi-site qualitative study involving in-depth interviews with bereaved parents. The analysis reported here examined parent experiences of the financial and employment impacts of their child receiving end-of-life care. A phenomenological approach guided all aspects of this study [22]. We report according to the Consolidated Criteria for Reporting Qualitative Research (COREQ) [23].

### Ethics approval

This study had UK-wide approvals from the Health Research Authority and Health and Care Research Wales (20/01/2022, 300913) and West of Scotland Research Ethics Service (21/WS/0170).

### Patient and public involvement (PPI)

The study team worked collaboratively with a Parent Advisory Panel of 12 bereaved parents throughout all stages of the research, from grant application through to dissemination, including being represented on the oversight committee. These parents had a diverse range of backgrounds and experiences. A bereaved parent (GW, co-author) provided an in-depth contribution to the design and delivery of the study including participating in this analysis.

### Setting and recruitment

Children with life-limiting conditions are frequently admitted to hospital within the last year of their life, with more than 70% of children in the UK dying in hospital settings [24], although admissions vary significantly across the type and number of conditions [25]. Within this context, four NHS hospital settings provide care for over 60% of children in their last year of life: principal treatment centres for cancer for children (C-PTC) or for teenagers and young adults’ (TYA-PTC), and neonatal and paediatric intensive care units (NICU and PICU respectively) [26–29]. Parents of children who received end-of-life care in one of these four NHS settings were the focus of the workstream [1]. 

The first workstream of the ENHANCE study identified the components of end-of-life care, and highlighted the different ways in which settings operationalised these and therefore how delivered care varied [1, 30]. To ensure a diverse sample, participant recruitment sites were selected based upon the need to capture this variation in provision. The Parent Advisory Panel, concerned about practitioner gatekeeping and aiming to broaden the scope of recruitment, advised recruiting via parent-facing organisations and social media platforms. Therefore, the study was also advertised via charity organisations’ social media.

At each site health professionals identified eligible parents and then discussed the study with them, either face-to-face or by telephone. Interested parents were given a brief information sheet, consent-to-contact form, and a pre-paid return envelope addressed to the study team. Once the study team received the consent-to-contact form, they sent the parent an invitation letter and a detailed information sheet either via post or email. The team then followed these up with a telephone call to answer any questions and arrange the interview. Interested parents who read about the study via social media, contacted the study team directly, either by telephone or email, and were then sent a letter and detailed information sheet and followed up, as above.

### Sampling

Purposive sampling was used to ensure representation across the UK, across the four settings, and according to child characteristics, including: diagnosis, age, and illness duration. These factors have been identified as potentially affecting access to end-of-life care [31]. 

Parents or legal guardians were eligible if they were aged 16 + years, and their child had received end-of-life care from a neonatal or paediatric intensive care unit, or a children’s or teenage and young adult cancer principal treatment centre in the UK. Parents of children or young people aged up to 25 were included to reflect that many paediatric services retain patients over 18, and to ensure that the experiences of end-of-life care in the teenage and young adult cancer treatment centres were captured. To be eligible, a parents’ child must have died in the last three years and not within the last three months; this criterion is based on findings in previous relevant research, the team’s experience of conducting research with bereaved parents, and input from our parent advisors [32, 33]. 

### Data collection

In-depth interviews explored bereaved parents’ accounts of end-of-life care for their child. Parents chose to be interviewed either face-to-face, by telephone or by video-call (ZOOM or MS Teams). Where both parents participated, they were offered either individual or joint interviews. Interviews were undertaken by four authors, all previously unknown to participants (EVM, LB, JH; all females; GP, male; all applied health researchers).

In the interview, parents were first invited to tell the story from their child’s diagnosis through to after their death using in their own words and in an unstructured and personal manner, enabling them to focus on the phenomenon and experiences that were important to them [22]. The second part entailed a more structured approach to identify and explore specific components of end-of-life care, identified in the first ENHANCE workstream, if not sufficiently captured in the parents’ story, including asking about any financial and employment impacts [1]. (see supplement 1 for outline topic guide). Interviews were audio-recorded and transcribed verbatim. To minimise the burden of participation, transcripts were not returned to parents for checking. Researchers debriefed after each interview and every two weeks as a group.

### Data analysis

Consistent with a phenomenological approach, data were analysed using thematic analysis to find and understand patterns of shared meaning from parents’ experiences of the financial impacts of their child receiving end-of-life care [34, 35]. The research team, including GW (co-author), met regularly to reflect and discuss the analysis. As part of the data familiarisation process, LB read and reread interview transcripts and then coded using a combination of inductive coding that stayed close to participants words to identify key concepts expressed and deductive coding using topic guide prompts. Codes were grouped into descriptive categories and then LB and JH iteratively developed analytical themes by seeking patterns of shared meaning. NVivo [14] was used for managing and analysing data [36]. 

### Reflexivity statement

Study team members were predominantly female, with a range of perspectives (health professionals, public health, policy, methodological) and different levels of experience in end-of-life care. External stakeholders provided additional perspectives and created a more balanced gendered team. To aid group discussions, increase transparency and inform perspectives on data interpretation, interviewers completed reflective journals following each interview and met every two weeks to discuss and reflect upon potential biases.

## Results

Bereaved parents were recruited via NHS sites (*n* = 14), parent-facing organisations or social media channels between Sept 2022 and July 2023. Forty-two interviews with fifty-five parents were conducted (Fathers = 16, Mothers = 39), representing 44 children. Thirteen were joint interviews with the mother and father, and two interviews concerned end-of-life care for two children. Mean interview length was 99 min (range: 49–221 min). Table 1 sets out the characteristics of the sample including child age and gender, parents’ gender and ethnicity and the place of death for the child. Also shown are the number and source referrals to a hospice for post death care and support (Table 1).

**Table 1:** Overview of sample characteristics

Age of child when died	Number
< 12 months	23
1–4 years	4
5–10 years	3
11–18 years	9
19–25 years	5
**Gender of child**	
Male	25
Female	19
**Gender of parent**	
Male	16
Female	39
**Ethnicity of parent**	
White British	52
British Asian	2
Unknown	1
Any other siblings*	
Yes	30
No	14
**Child’s place of death**	
Hospital setting	29
Home	6
Hospice	9
**Hospice referral post-death**	
Via NICU	1
Via PICU	3
Via PTC	0

*Relates to whether the child had siblings at the time of the death, not at the time of data collection

## Themes

Four themes were developed: (1) The added cost burden; (2) Pressures of juggling work; (3) Accessing support; and (4) Financial impacts continue after a child dies.

### 1) The added cost burden

Parents reported numerous direct out-of-pocket expenses associated with having their child admitted to hospital. Expenses included travel, food and accommodation and some families were left worrying about how they were going to cover their bills “*we really*,* really*,* really did struggle for money” (16: mother*,* NICU).*

Only a very few parents were provided with meals on wards, the others were left to fend for themselves. Most found that hospital food was expensive, low quality and offered limited choice “*the hospital food’s crap*” *(51: father*,* C-PTC)*. The pressures of having a child in hospital at the end-of-life, and the need to spend time with them meant many families were out of their daily routines, and were not weekly budgeting, regular meal planning or bulk shopping, with one parent describing this as running two households.

*I can’t even tell you how much money I spent on food*,* I feel disgusted thinking about it. (18: mother*,* PICU)*

In order to manage, parents discussed trying to get themselves as organised as possible, doing online food shops and batch cooking.

*For us to be able to get food we were having to order it to our home address and [Father] was having to drive there and just wait for a Tesco delivery and just put it straight in the car. (23: mother*,* NICU)*

Families who were not offered accommodation nearby or had to be at home with other siblings, commuted to the hospital daily, meaning that there were additional costs such as rail fares, petrol and parking costs. For parents of babies, the mothers were often provided with beds on the ward, leaving the fathers to make the journey to and from the hospital alone. Families talked of petrol bills costing over £60 a week, and parking was reported to have cost some families up to £25 a day.

Parents highlighted that it was not just extra financial costs they paid, there were also opportunity costs. For example, when parents had to leave the wards to go and find food either from the hospital canteen or local shops, this was time away from their child. Parents resented a system where the expenses and practicalities of having a sick child and having to ‘live’ at the hospital added extra sources of stress to a difficult situation.

*And the one thing you don’t want to be worrying about is money*,* it’s crazy that even has to be in your mind. […] I don’t know if you can tell but I am still angry about that. I think it’s fair to be angry because they made it that little bit harder and another worry. (11: mother*,* C-PTC)*

### 2) Pressures of juggling work

Most parents found that they needed to rearrange their working hours to some extent so that they could prioritise spending time with and caring for their child. *“I stopped working as soon as we found out because obviously [.] it was like the least of my worries.” (11: mother*,* C-PTC)* This involved reducing working hours, taking leave or having to quit jobs. The financial repercussions of parents working less added to the pressures of the situation.

*We are really struggling financially because of this*,* obviously [Father] was having to take a lot of time off work to come to meetings and appointments with me. This has had a massive financial burden on us. (03: mother*,* NICU)*

In many families, the onus to keep working to cover bills, rent or mortgages, as well as covering the extra costs fell to one parent, often fathers, either by explicit agreement or due to employment circumstances such as being self-employed. All parents juggled their time as well as they could, including working extra days, in the evenings and even by the child’s bedside. However, maintaining performance at work was difficult, and several parents felt the added worry that they were letting their employers and colleagues down as their focus was elsewhere.

*The whole work thing unravelled*,* […] I was sort of working here and there and putting pressure on other people. (34: mother*,* TYA-PTC)*

Employers’ support to the family’s situation, in terms of time off (paid or unpaid) and pressure to return to work, made a big difference to parents’ experiences. Parents described a real range of responses, and these were often dependent on the attitudes of individual managers with little consistency in how policies were applied. When parents were supported in terms of job security and clarity around income, they experienced less stress and were able to focus their attentions on their child.

*So obviously*,* the whole time this was happening*,* I was still getting paid*,* which is good because if I wasn’t*,* it would have been a lot harder. (16a: father*,* NICU)*

Other parents however faced a tougher situation and felt unsupported by their employers “*I was working but*,* no*,* no help from work at all.” (35: mother*,* NICU)*, they had to battle for time off, often taking unpaid leave and in one case losing their job.

*Because my husband was working for a really shitty company as well at that time and they didn’t give him paid paternity leave or anything […] No*,* he couldn’t take annual leave*,* it had to be unpaid leave. His boss was awful. And then he made him redundant just after [Child] died. Awful man. (08: mother*,* PICU)*

The COVID-19 pandemic enabled some parents to work remotely, and in a few cases, parents were specifically put on furlough to allow them to be with their child.

*They’d [employer] purposely put him [Father] on furlough so that he could spend some more time with [Child]*,* and I was so grateful because when [Child] passed that would’ve been a normal workday for him and he wouldn’t have been there that day*,* he would’ve been at work. So just for us to be together and to have spent so much time together as a family was just incredible*,* I can’t thank them enough for that. (50: mother*,* PICU)*

For parents of new babies, the disparity in maternity and paternity entitlements had profound impact on how mothers and fathers were able to choose and plan to be with their child. Fathers reported only getting two weeks paternity leave, and when faced with uncertainty about their child’s future did not always know how best to use that time. For one family, a father delayed using his leave as he hoped to use it when the child left hospital and so missed spending time with his child before they died.

*Whereas for [Father] it was quite different because he was only entitled to two weeks paternity leave*,* which he was thinking*,* “Well I’ll want that two weeks’ paternity once we bring our baby home.” (23: mother*,* NICU)*.

### 3) Accessing support

Hospitals offered practical and financial support to many families, and most commonly direct assistance took the form of parking permits, meals or food vouchers and accommodation. However, it was delivered in an ad hoc and inconsistent manner; parents described a lack of transparency and challenges navigating what was available, as well as variability of entitlement criteria and piecemeal delivery.

*I know there are a few things out there*,* but they’re not necessarily passed on to the parents to tell them actually you can use this*,* or you’ve got that. (52: mother*,* TYA-PTC)*

Several families spoke of direct financial support received from charities including one-off payments from the hospital trust charities. Charities also funded accommodation at some hospital sites, enabling families to be nearby. Charity funded posts were often in place alongside NHS staff, particularly in oncology teams, providing family support, liaison and playworker roles. However, it was not clear to some families about the specific role of these workers within an integrated team, including what support was available and by whom.

*That was somebody*,* she was almost like a Family Liaison person in the hospital. I have a feeling*,* but I might be wrong*,* that she worked for the Children’s Heart Foundation*,* like a charity within the hospital. They did things like gave us meal vouchers to use at the canteen. (07: mother*,* PICU)*

The variability of additional financial and practical support provided, and patchiness of information about entitlement added to some families’ feelings of resentment - it felt unfair when they saw other families getting support while they were struggling.

*I’ve never received anything*,* and I never knew I was entitled because they never told me*,* it could have helped so much. […] I had no idea and everybody else was like getting days out or getting grants or disabled badges*,* like anything to make it easier for them and we had zero*,* absolutely zero and that really bothers me because how unfair is it? (11: mother*,* C-PTC)*

Despite potentially being entitled to government financial support through social security benefits, families that tried to navigate the claims system found the process daunting, complicated and slow, with long delays in decision making. In a few cases parents mentioned that a social worker helped them, and cancer specific charities such as CLIC Sargent and Macmillan were highlighted as providing valuable signposting and help with form filling.

*They were brilliant and they were able to help me with the financial side of things*,* CLIC Sargent* [now Young Lives vs. Cancer] *sorted out all of the forms that needed to be done and they went through it all with me and ensured that I got what I could*,* what I was entitled to*,* which really did help because I was self-employed (52: mother*,* TYA-PTC )*.

Ultimately, for many parents the main source of practical assistance came from family, friends and colleagues rallying round; they provided a whole raft of help including cooking food, babysitting for siblings and lifts to and from the hospital “*your mum was so amazing and cooked lasagnes for us.”* (19: mother, NICU) Several parents spoke of friends setting up GoFundMe pages, and there were many reports of community fundraising efforts such as bike rides and sponsored walks, as well as direct donations.

*So every week the guy who was covering for [Father] would give us this envelope of money and that is literally what got us through and we were able to pay the bills on top of the days that [Father] was working; if it wasn’t for them we would be so effed*,* so badly*,* (11: mother*,* C-PTC)*

### 4) Financial impacts continue after a child dies

For parents, the financial impact of their child’s end-of-life care continued after they had died. Immediate concerns about paying for funerals were to some extent alleviated by the grant from the Children’s Funeral Fund, although this did not cover all the expenses such as headstones.

*So financially that’s taken care of*,* yeah*,* and I think that’s important. I think the government do pay for under-16s. I think there were a few things that we paid for*,* but yeah. I think that’s good. (08: mother*,* PICU)*

Many employers continued to be supportive of bereaved parents and gave them space, and time off to grieve without the pressure of returning for work. For most of these parents, the time came when they were ready to go back to work, and those that were then able to gradually ease back in, appreciated flexibility.

*And then returning to work*,* you know*,* my boss was really*,* really supportive. I returned on a gradual phased return. (23: mother*,* NICU)*

However, for families where there was more financial pressure to return to work, this often fell on fathers and was exacerbated by lack of compassionate leave - statutory entitlements of bereavement leave is only two weeks. This meant that many fathers were returning to work very quickly after their child’s death, with little if any bereavement support.

*And financially there needs to be more out there for dads to grieve too. There isn’t any time for him to have off work and have that financial burden on his shoulders. (03: mother*,* NICU)*

Settling back in to work for many had been difficult and, in some cases, impossible. Parents described being unable to concentrate, having a short fuse and finding it difficult to know how to discuss their experiences with colleagues. Other parents, often mothers, who had given up work to care for their child, found themselves jobless, with the daunting task of re-entering the workplace.

*The thought of me going to an interview makes me feel physically sick*,* how would I…? What if they ask me? Is it going to come up*,* what if they ask? you know*,* I was so worried. (21: mother*,* C-PTC)*

Families who were in receipt of disability living allowance quickly found they were no longer eligible for this financial support once their child had died. The swiftness of the system to recognise the change in circumstances and stop payments was an added shock especially given the lengthy delays they faced when making their application for support.

*And 2 days after [child] died*,* I got a letter to say that my Child Disability Living Allowance stopped*,* like how can they process it that quick*,* but it took 18 weeks to set up and I didn’t get a penny of back pay? (11: mother*,* C-PTC)*

## Discussion

Parents’ experiences of the direct and indirect financial consequences of a child receiving end-of-life care have been identified in this study. The effects include the costs of running two homes if a child is in hospital, the pressures of retaining a job and issues with accessing financial support. The financial repercussions continue after the death of a child and the cumulative effect on families should not be underestimated, with some families having to manage this impact for many years.

This study was the first piece of UK academic research for about twenty years that aimed to understand the financial impacts on a family when a child dies, and parents experiences have hardly changed [15]. More recent studies have tried to quantify the financial impact of having a child with a disability [37], the cost to a family of a critical care admission and the cost of their child receiving a cancer diagnosis [10, 38, 39] but these do not take into account the full and lasting financial impacts of having a child receiving end-of-life care and then dying, highlighted here.

In order to reduce the immediate cost burden, subsidised food and accommodation is often available, either directly from the ward or via charities [39], but this support needs to be more proactively and systematically communicated to families by their healthcare providers. Government policy on free car parking should also apply to these families, however, the government acknowledges this is not always feasible due to the lack of parking spaces [40]. Whilst charities have been shown to try to plug some of the gaps [41], this support, as reported in the current study, is often ad-hoc, inconsistent and confusing about who can access it and how.

The inadequacies of the state benefit system in the UK to support families of children with disabilities and life-limiting conditions have been raised repeatedly, including recently in relation to the cost of living crisis and energy prices [42, 43]. Parents with a child at the end of life qualify for government financial support, such as Disability Living allowance, Carers Allowance and Universal Credit [44]. Many families in this study were unaware of their rights, and for those that had applied, they experienced long delays, and in some cases the parents simply could not face the application process. This may be particularly true when it is an infant who is seriously unwell or dies [14, 45], and there is also evidence of low uptake of benefit payments in some minoritised ethnic families [46]. Recent research by a children’s cancer charity found that families wait on average seven months between a diagnosis and a decision on their disability benefits [10]. Transparency and clear signposting to specific professionals responsible for supporting families to navigate the benefits system, would mean that more families receive timely financial support to which they are entitled. The rapidity with which some benefit payments, in particular Disability Living Allowance, cease after the death of a child should be questioned, given that the financial impacts on families continue. A transition to a bereavement support would acknowledge a family’s change in circumstances, while providing some financial cushioning.

The ‘opportunity cost’ of time spent working, trying to access support, or travelling to and from the place where the child was being cared for, was that parents were not spending time with their child. Bereaved parents in this study resented missing out on time with their child before they died, and this could potentially have longer-term implications for their grief outcomes [47]. The introduction of Neonatal Care Leave will be a welcome support for parents of babies [48]. Being together as a family, memory making and enabling parents to fulfil their parental role as their child dies, has previously been identified as key to delivering high quality end-of-life care [49, 50]. 

In general, free bereavement support for parents is lacking in both availability and appropriateness to meet their specific needs and very rarely does it address employment issues [6, 51]. It is likely to be beneficial for society, as well as families themselves, for parents to have the opportunity to rejoin the workforce when and how it is right for them. Stroebe and Schut’s Dual Process Model of Grief [52] underpins the importance of providing supportive and flexible approach to returning to work, so bereaved parents can try to balance loss-orientated processing with restoration orientation tasks.

While employees are entitled to two weeks of parental bereavement leave [53], parents in the study felt this was inflexible and wholly inadequate, and those who are self-employed are not covered. Guidance for employers on providing more tailored support for bereaved parents to return to their jobs is scarce and this study has shown that bereaved parents are very dependent on individual employers’ compassion, policies and practices, which are ad-hoc and inconsistent. This study also highlights how a primary carer of a child who has died, following a life-limiting condition, may have been out of paid employment for many years, and so will also require targeted training and support to join the workforce.

### Strengths and limitations

The study benefited from being a large national study which included the experiences of a range of parents and children. The qualitative approach enabled a deep understanding of the parents’ perspectives of the financial implications, but less ability to quantify that impact. Due to the interviews being undertaken within a relatively short period of the child’s death, the longer-term implications on a family’s finances and employment prospects, and on wider society, have not been fully explored and so could benefit from further research. Although represented in this study, there is scope for further research about the specific impact on fathers [54–56], in particular around the pressures to return to working and the decisions about to when to use limited leave. However, this study provides insight to inform the first steps to making a change for the better.

## Conclusion

Financial hardship is a known consequence of having a child with a life-limiting condition, especially at the end of life, and adds considerable stress to an already painful situation, with the aftermaths continuing into bereavement. The impact of the significant additional costs is exacerbated by parents’ need to reduce work so they can spend time caring for their dying child, and this leaves families in a financially and emotionally vulnerable position. Health and social care professionals have a responsibility to signpost parents to the financial support they are entitled to. There needs to be a transparent and consistent approach to immediate practical support from healthcare providers; a review of benefit system delays and the abrupt stopping of Disability Living Allowance; and development of tailored support for retaining and rejoining the workforce.

## Electronic supplementary material

Below is the link to the electronic supplementary material.

Supplementary Material 1

## Data Availability

The dataset generated and analysed during the current study, in the form of interview transcripts, are not publicly available due to ethical considerations (to ensure data confidentiality and protect the anonymity of the research participants).
