# Supplementary material for: Parents’ experiences of the financial and employment impacts of their child receiving end-of-life care: a national qualitative study
Source: BMC Palliat Care. 2025 Jun 4;24:157. doi: 10.1186/s12904-025-01796-1 (PMC12139220; doi:10.1186/s12904-025-01796-1)
Supplement: Supplementary file 1 — Supplementary Material 1 [file 12904_2025_1796_MOESM1_ESM.docx]

**End of Life Care for Infants, Children and Young People** **(ENHANCE)**

Workstream 2: to investigate the experiences and outcomes for children, parents and health professionals of different models of providing end of life care

**Outline Topic Guide –Parents - Interviews**

**Outline of structure**

**Introducing the interview and consenting process**

**Finding out about the child and family**

**History of the child’s condition**

**The parent’s story of their experiences of palliative and end of life care for their child**

**Semi-structured follow-up questions on target topic areas, if not covered in parent’s story:**

**Care in the last days, weeks and months of life**

**Employment, working arrangements and financial impact**

Example prompts: What was your work life during this period? During this time, did you experience any kind of impact on your finances? Can you tell us about any costs or expenses you might have had during this time? What, if any financial or practical support was offered to you during this time?

**Learning from what works less well**

**Learning from what works well**

**Suggested improvements and final thoughts**

**Close**
